# Odd–Even Effect in Peptide SAMs—Competition of Secondary Structure and Molecule–Substrate Interaction

**DOI:** 10.1021/acs.jpcb.1c06625

**Published:** 2021-09-23

**Authors:** Agnieszka Grabarek, Łukasz Walczak, Piotr Cyganik

**Affiliations:** † Smoluchowski Institute of Physics, Jagiellonian University, Łojasiewicza 11, 30-348 Krakow, Poland; ‡ Science & Research Division, PREVAC sp. z o.o., Raciborska 61, 44-362 Rogow, Poland

## Abstract

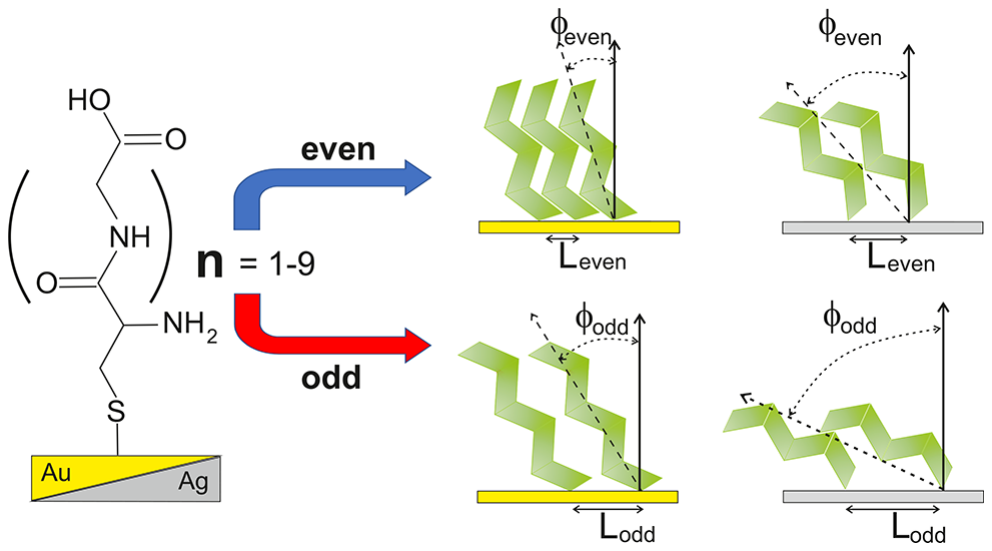

Peptide-based self-assembled monolayers (SAMs) are well known to be crucial for biocompatible surface formation on inorganic substrates applied for implants, biosensors, or tissue engineering. Moreover, recently these bioinspired nanostructures are also considered particularly interesting for molecular electronics applications due to their surprisingly high conductance and thickness-independent capacitance, which make them a very promising element of organic field-effect transistors (OFETs). Our structural analysis conducted for a series of prototypic homooligopeptides based on glycine (Gly) with cysteine (Cys) as a substrate bonding group chemisorbed on Au and Ag metal substrates (Gly_*n*_Cys/Au(Ag), *n* = 1–9) exhibits the formation by these monolayers secondary structure close to β-sheet conformation with pronounced *odd–even* structural effect strongly affecting packing density and conformation of molecules in the monolayer, which depend on the length of molecules and the type of metal substrate. Our experiments indicate that the origin of these structural effects is related to the either cooperative or competitive relationship between the type of secondary structure formed by these molecules and the directional character of their chemical bonding to the metal substrate. The current analysis opens up the opportunity for the rational design of these biologically inspired nanostructures, which is crucial both for mentioned biological and electronic applications.

## 1 Introduction

Self-assembled monolayers (SAMs) provide a simple and robust way for controlling an organic–inorganic interface by the formation of a predefined, ultrathin, and stable organic film on inorganic substrates.^1,2^ The precise control of such interface on metal substrates becomes currently mandatory in two vast areas of applications, *i.e.*, molecular/organic electronics^3−6^ and biocompatible coatings.^1,7−9^ On the one hand, formation of such biocompatible coatings on metal substrates by SAMs is driven by their application for implants,^7^ tissue engineering,^9^ or biosensors.^10^ On the other hand, ultrathin organic films on a metal substrate formed by SAMs with well-defined conductivity, which is either high (for drain/source type electrodes)^11−13^ or low (for gate type electrode),^14,15^ are of fundamental importance for controlling charge transport across the metal-organic junctions in molecular and organic electronics devices.^3−6^ These two important areas of SAM applications are usually covered by structurally different types of monolayers. However, the recent analysis of peptide-based SAMs indicate that this type of thin, bioinspired, monolayers is potentially interesting not only in the area of biocompatible coatings^16,17^ but also in the field of molecular/organic electronics.^18−20^ In particular, high conductance of peptide-based SAMs was reported,^18−20^ which is close to “conductive” oligophenyl-based monolayers^11^ of comparable length and proceeds *via* superexchange-mediated tunneling,^18−20^ which probably involves interactions among high-energy occupied orbitals in multiple, consecutive amide bonds. Recent experiments^21^ show that peptide-based SAMs exhibit large capacitance which, surprisingly, is also thickness-independent and therefore very attractive for field-effect transistor applications, where low operational voltage of the transistors demands high capacitance of the gate junction. The charge transfer in peptide-based SAMs is strongly affected by the peptide chemical structure, conformation, and molecule–electrode contact.^20,22^ Therefore, electronic properties of peptide-based SAMs crucially depend on the conformation of peptide molecules in the monolayer, which is controlled by intermolecular, intramolecular, and molecule–substrate interactions.

**Figure 1:**
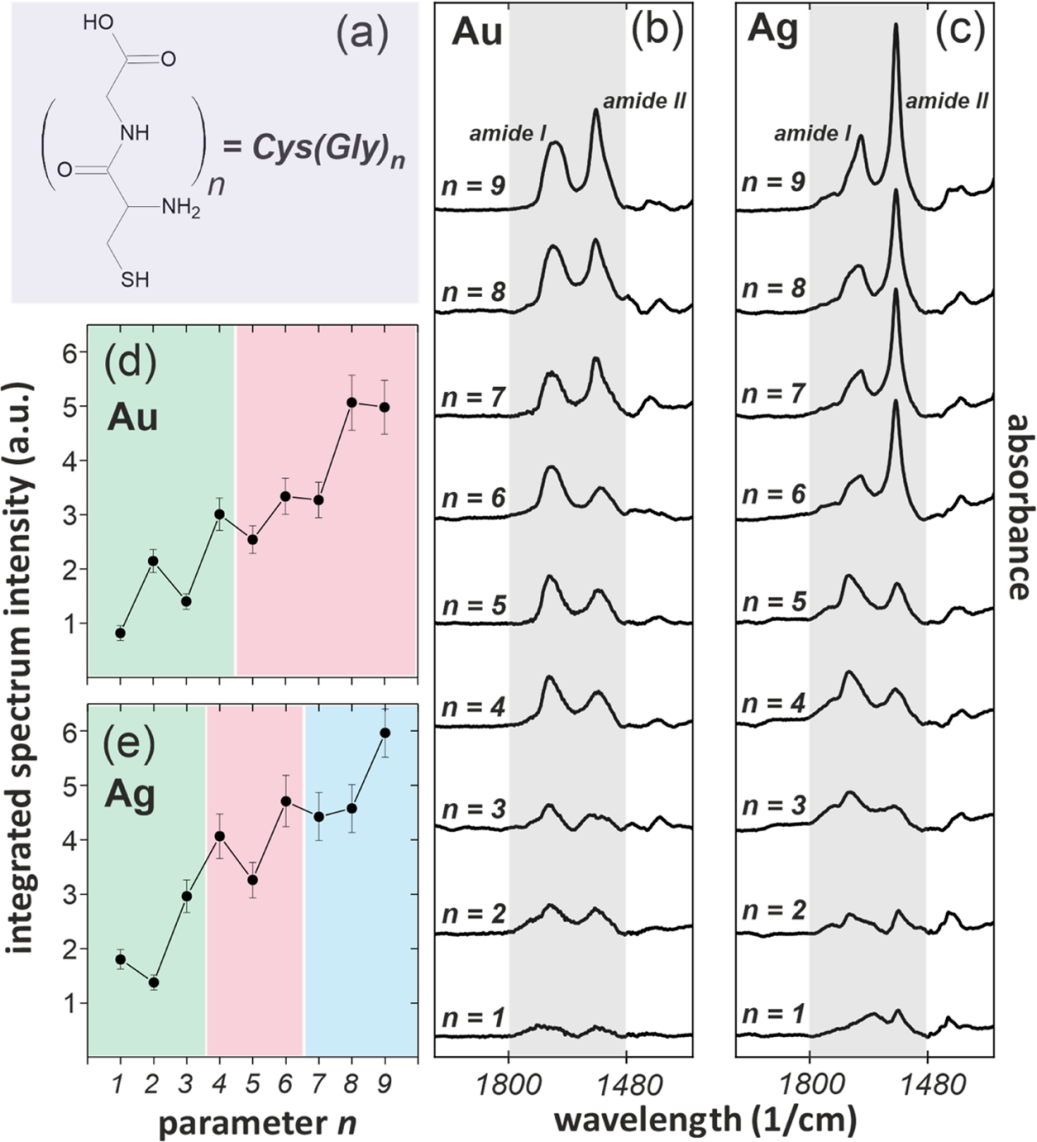
(a) Schematic presentation of the molecules used in this study and their acronyms. Panels (b) and (c) show IRRAS data for (Gly)_*n*_Cys/Au and (Gly)_*n*_Cys/Ag SAMs, respectively. The gray bands in (b) and (c) mark the range of IRRAS signal integration. Panels (d) and (e) integrated the IRRAS spectrum in the range of 1800–1480 cm^–1^ as a function of parameter *n* for (Gly)_*n*_Cys/Au and (Gly)_*n*_Cys/Ag SAMs, respectively (see the text for details).

To this end, in the current contribution, we correlate the impact of all of these interactions with the final confirmation of peptide-based SAMs on Au and Ag substrates using a model system of homooligopeptides based on glycine (Gly) with cysteine (Cys) as a substrate bonding group in the form of (Gly)_*n*_Cys/Au(Ag), where *n* = 1–9 (Figure 1a). Our results reveal that the structure of these SAMs, formed on two metal substrates (Au, Ag) most commonly used in experiments related to biocompatibility and molecular/organic electronics, depends on parameter *n* showing an *odd–even* effect. The appearance, magnitude, and phase of this *odd–even* effect depend both on the length of the molecule building monolayer and the type of the metal substrate. Current experiments indicate that the origin of these structural effects is related to the cooperative or competitive relationship between the type of secondary structure formed by these molecules and the directional character of their chemical bonding to the metal substrate.

## 2 Methods

### 2.1 (Gly)*_n_*Cys SAM Formation

The SAMs were formed on Au(111) and Ag(111) substrates prepared by evaporation of 100 nm of silver or gold onto single-crystal silicon (100) wafers (ITME, Warsaw) primed with a 5 nm chromium adhesion layer (base pressure of ∼10^–7^ mbar, rate 0.1 nm/s). Monolayers were formed by incubation (24 h) of Au or Ag substrates in 1 mM solutions of a given Cys(Gly)*_n_* compound (purity > 95% synthesized by Pepmic Co., Ltd., Suzhou, China) in deionized water, which has been refluxed and subsequently distilled under the flow of argon for 1 h directly before application with a solvent still system. The solutions were prepared in an argon atmosphere (<1 ppm O_2_) in a glovebox (MBRAUN). After incubation, samples were rinsed with deionized water to remove physisorbed molecules and dried under the N_2_ stream.

### 2.2 Spectroscopic Analysis by IRRAS

The Infrared reflection–absorption spectroscopy (IRRAS) measurements were collected with a Thermo Scientific Nicolet 6700 FTIR spectrometer (dry-air-purged) using an MCT detector and the p-polarized light incident at a fixed angle of 80° (with respect to the sample normal). All spectra (resolution 2 cm^–1^) are reported in absorbance units *A* = −log *R*/*R*_0_, where *R* is the reflectivity of the measured sample, whereas *R*_0_ is the reflectivity of the reference sample (substrate covered with perdeuterated hexadecanethiolate SAMs).

### 2.3 Spectroscopic Analysis by XPS

The X-ray photoelectron spectroscopy (XPS) measurements were performed with a UHV system by PREVAC sp. z o.o. with a monochromatized Al Kα source (*E* = 1486.6 eV, MX-650 VG Scienta) and a VG Scienta R3000 hemispherical analyzer with the base pressure in the analytical chamber at the level of ∼5 × 10^–9^ mbar. All spectra were collected at the normal emission geometry with the energy step of 0.15 eV and the overall energy resolution estimated at ∼1.15 eV, based on the full width at half-maximum (fwhm) of the Au 4f_7/2_ peak, which was also used as a reference for the binding energy (BE) scale (BE for Au 4f_7/2_ = 84 eV). The photoemission peaks were fitted using the Voigt profile after subtraction of the inelastic background fitted with the Shirley method. To fit S 2p_3/2,1/2_ doublets, a pair of peaks with a branching ratio of 2:1 (p_3/2_/p_1/2_) and spin-orbit splitting of ∼1.18 eV was used.

## 3 Results and Discussion

**Figure 2:**
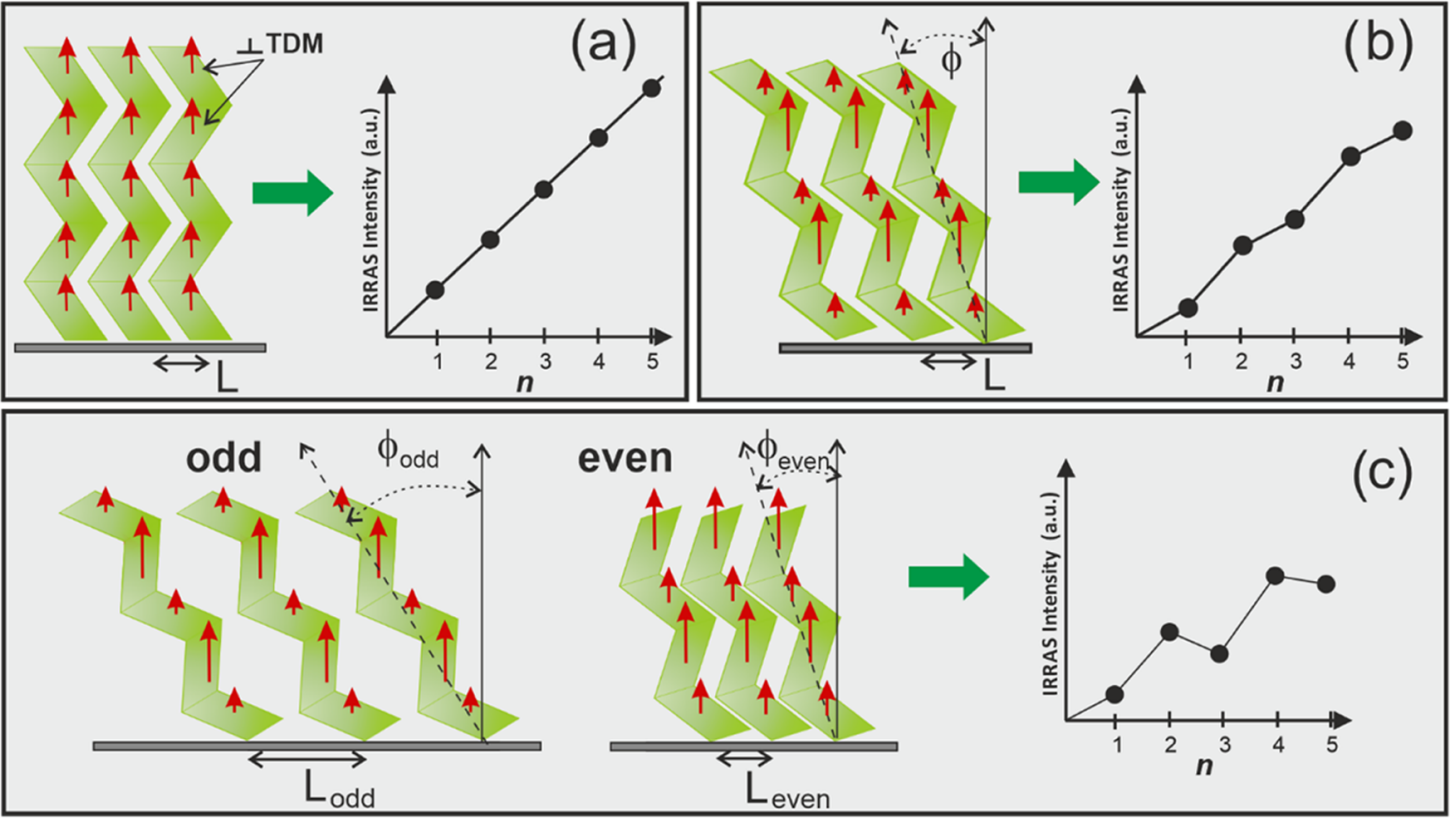
Schematically presented a change of the integrated intensity of the IRRAS spectra for (Gly)_*n*_Cys (*n* = 1–5) as a function of the parameter *n* for three different structural models: (a) normally oriented β-sheet structures with constant distance *L* between structures for all members of the series, (b) tilted β-sheet structures with a constant value of the tilt angle ϕ and the distance *L* for all members of the series, and (c) tilted β-sheet structures with tilt and distance between structures different for *odd* (ϕ_odd_, *L*_odd_) and *even* (ϕ_even_, *L*_even_) members of the series. The red arrows in structural cartoons shown in (a)–(c) mark the normal component of TDM associated with an arbitrary selected vibration within the Gly unit of the β-sheet structure.

The (Gly)_*n*_Cys/Au(Ag) SAMs were prepared from respective deionized and degassed water solutions at room temperature in the argon atmosphere. Spectroscopic characterization of monolayers was conducted using infrared reflection absorption spectroscopy (IRRAS) and X-ray photoelectron spectroscopy (XPS). The summary of the IRRAS analysis is presented in Figure 1. To determine the conformation of molecules, we have limited our analysis to the frequency range (1300–2000 cm^–1^), which covers absorption band characteristic for peptides and related to C=O stretching and N–H bending vibrations described as *amide I* and *amide II* bands, respectively. For both types of SAMs, two absorption bands are visible in this frequency range. The lower frequency band, with the maximum located at ∼1553–1565 cm^–1^, is consistent with the *amide II* range. The maximum of the higher frequency band is located within the *amide I* range, and its position is indicative of the peptide secondary structure. For (Gly)_*n*_Cys/Au SAMs, the maximum of the *amide I* band is located at ∼1675–1690 cm^–1^, which is characteristic for β-sheet type of the secondary structure.^23,24^ However, for longer members of the (Gly)_*n*_Cys/Ag series with *n* = 6–9, the position of this band is shifted toward lower frequencies at ∼1658 cm^–1^, which may suggest the formation of some looped structures close to α-helical conformation.^23,24^ Considering that every Gly unit contributes to the total absorption measured for the given (Gly)_*n*_Cys monolayer within the *amide I* and *amide II* bands, we may expect a general increase in the total intensity of the measured signal in this range with the number *n*. To make such analysis reliable, we have calculated an integral over the spectra in the range 1480–1800 cm^–1^ (indicated in Figure 1b,c) and plotted it as a function of parameter *n* (Figure 1d–e). For (Gly)_*n*_Cys/Au, the result of this simple analysis exhibits not only an overall growth of the measured *amide I* and *amide II* intensity but, more importantly, a pronounced *odd–even* effect with higher intensity measured for the *even* members of this series. We note that for molecules on a metal substrate the intensity of the given absorption band measured by IRRAS depends on the orientation of the respective transition dipole moments (TDMs) with respect to the metal substrate, and only normal (to the metal substrate) component of the TDM is detected following surface selection rules (SSR).^25−27^ Therefore, such *odd–even* effect that can be visible in IRRAS, but not in regular IR bulk data, is a fingerprint of the secondary structure formation by the homologue oligopeptide (Gly)_*n*_Cys/Au series. To correlate the observed *odd–even* effect with the possible β-sheet secondary structures formed by the (Gly)_*n*_Cys molecules on metal substrates, we have considered three simple structural models. The first model (Figure 2a) assumes normal orientation of β-sheet secondary structures and constant distance *L* between them (and hence constant surface packing density) for all members of the series. In such model, symmetric orientation of *odd* and *even* segments of β-sheet structures, relative to the surface normal, precludes the *odd–even* variation in the IRRAS data due to the equal magnitude of the normal component of TDM vector, associated with any particular vibration within the analyzed range (schematically indicated in respective schemes by red arrows), for *odd* and *even* segments. The *odd–even* change in the integrated IRRAS signal can be observed, however, assuming for all members of the (Gly)_*n*_Cys series fixed distance *L* and fixed tilt angle ϕ relative to the substrate normal, which breaks the symmetry between contributions from *odd* and *even* segments of β-sheet structures (Figure 2b). However, in contrast to our experimental data, the *odd–even* change in this model cannot lead to the lower IRRAS intensity for the longer member of the (Gly)_*n*_Cys series. Instead, a continuous growth of the integrated IRRAS signal with parameter *n* should be observed (Figure 2b). To mimic experimentally observed *odd–even* oscillation, we have to assume that the tilt angle ϕ and the distance *L* between β-sheet structures have different values for *odd* and *even* members of the series, *i.e.*, ϕ_odd_/*L*_odd_ and ϕ_even_/*L*_even_, respectively (Figure 2c). This observation not only confirms formation by the (Gly)_*n*_Cys/Au SAM secondary structure which is close to the β-sheet conformation, but more importantly, it shows an *odd–even* structural effect that impacts both the orientation and packing density of the molecules. Moreover, the green and red backgrounds in Figure 1d mark two different ranges of the amplitude of this *odd–even* effect for short (*n* = 1–4) and long (*n* = 5–9) (Gly)_*n*_Cys/Au SAMs. This observation reflects different tilt and packing densities of β-sheet-like structures for shorter and longer molecules within this series. Such change in the tilt of molecular axis between shorter and longer molecules building SAMs has been observed even for simple alkanethiols^28^ and can be attributed to increased intermolecular interactions with the length of the molecular backbone.

For (Gly)_*n*_Cys/Ag, the *odd–even* behavior in IRRAS data is much more complicated, *i.e.*, the phase of this effect is changing along with the series, and three different ranges can be defined (green, red, and blue background in Figure 1e). For *n* = 1–3, the phase of this effect is reversed compared to (Gly)_*n*_Cys/Au. Such behavior is very similar to the *odd–even* effect for biphenyl-substituted alkanethiols BP*n*S/Au(Ag) SAMs (CH_3_–C_6_H_4_–C_6_H_4_–(CH_2_)_*n*_–S/Au(Ag), *n* = 1–6) on Au and Ag substrates and originates from different bonding geometries of sulfur on both substrates, *i.e.*, the sp^3^-like and *sp*-like geometry on Au and Ag, respectively.^27,29^ This change of the preferred bonding group configuration reverses the phase of the β-sheet structure. However, for *n* = 4–6, the phase of the *odd–even* effect is reversed again and consistent with (Gly)_*n*_Cys/Au. This can be assumed considering that the energetic gain from preserving the preferred bonding group configuration is becoming less and less important for the increased length of the molecule, where the contribution of intermolecular interactions dictates the overall energetics of the monolayer. This observation suggests that the phase of the *odd–even* effect observed for (Gly)_*n*_Cys/Au provides the energetically favorable structure of such peptide SAMs, which is also adopted by longer members of the (Gly)_*n*_Cys/Ag series despite different bonding group configurations. Surprisingly, for the longest members of the (Gly)_*n*_Cys/Ag series with *n* = 7–9, the phase and amplitude of this *odd–even* effect are changed again. This implies that different secondary structures are adopted in this case, which is consistent with the significant shift of the *amide I* band, indicating the formation of a looped configuration. The looped secondary structure formation for the longest members of the (Gly)_*n*_Cys/Ag series might result from possible chemical bonding of the top carboxylic group with the Ag substrate.

**Figure 3:**
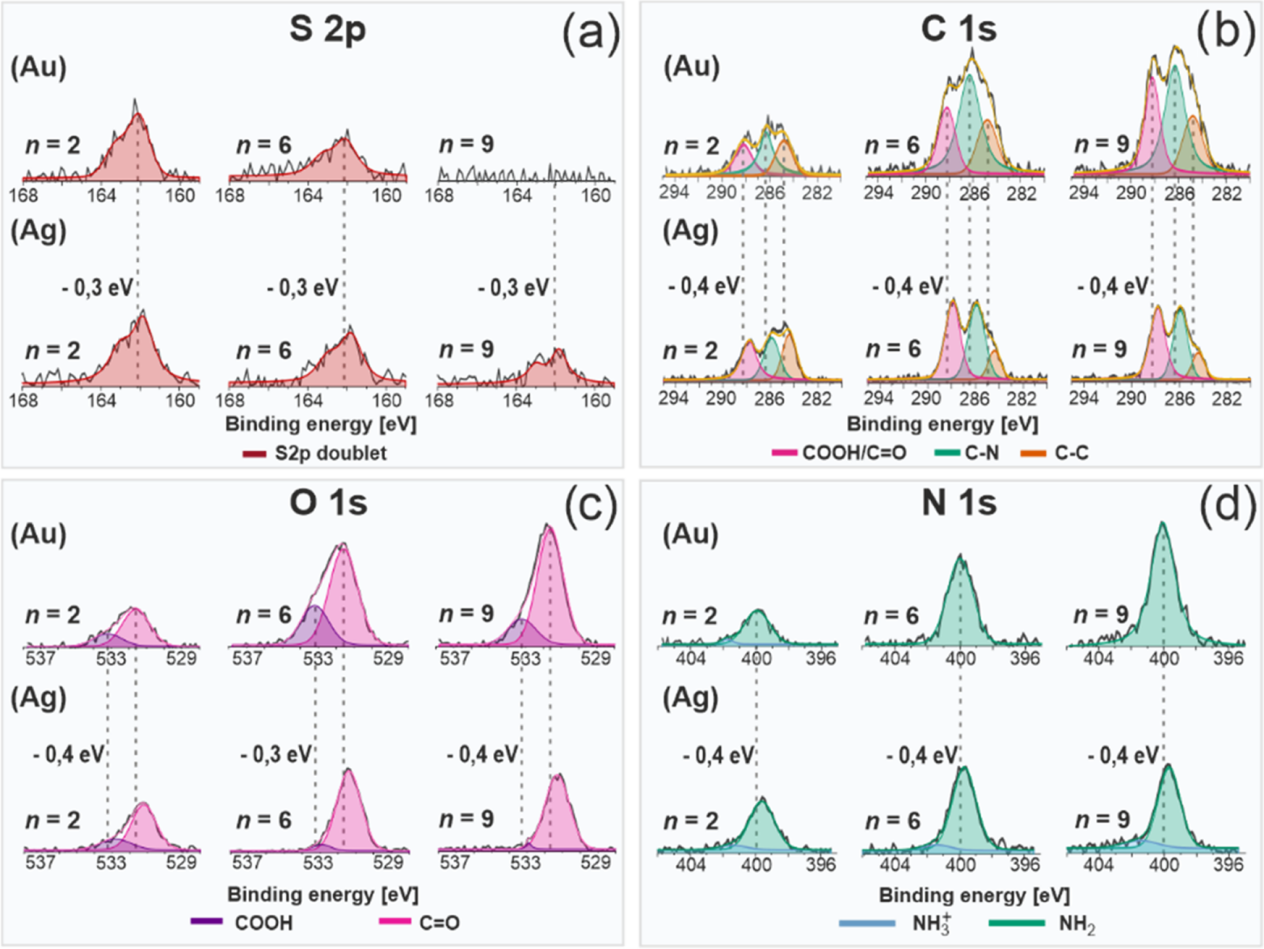
XPS data for (Gly)_*n*_Cys/Au (upper part of panels) and (Gly)_*n*_Cys /Ag (lower part of panels) SAMs with *n* = 2, 6, and 9 in the binding energy range of S 2p (a), C 1s (b), O 1s (c), and N 1s (d) signals. The dashed lines mark the position of maximum of the given signal component obtained for (Gly)_*n*_Cys/Au SAMs (see the text for details). Next to the signal peaks obtained for (Gly)_*n*_Cys/Ag SAMs, the value of the binding energy shift is provided.

The XPS spectra obtained for short (*n* = 2), intermediate (*n* = 6), and long (*n* = 9) members of the (Gly)_*n*_Cys/Au series are presented in Figure 3, as a representative example. The S 2p signal consists of a single doublet with the S 2p_3/2_ peak centered at binding energy (BE) ∼162 eV, which confirms well-defined Au–S bond formation, with no visible contribution of atomic sulfur or other thiolate species (Figure 3a).^18,21,30^ The intensity of the S 2p signal drops with an increased length of the (Gly)_*n*_Cys molecule, and for *n* = 9, it is below the detection limit of our XPS system (at a given acquisition time) due to the increased film thickness (*vide infra*) and the resulting attenuation of this photoelectron signal. The C 1s signal (Figure 3b) was fitted using three main components corresponding to C=O/COOH (∼288.2 eV), C–N (∼286.3 eV), and C–C (∼284.8 eV).^18,21,30,31^ The O 1s signal (Figure 3c) can be fitted with two components that correspond to C=O (∼531.6 eV) and COOH (∼533.2 eV) groups.^18,21,30^ The data obtained for the N 1s region (Figure 3d) show the main peak characteristic for −CONH and −NH_2_ groups (∼400.0 eV) and the minor high-energy component (∼401.8 eV) associated with the NH_3_^+^ zwitterion formation at the bonding cysteine unit,^18,21,30,31^ which is visible only for short members of the (Gly)_*n*_Cys/Au series due to the signal attenuation for higher film thickness (*vide infra*). The analogous XPS data obtained for (Gly)_*n*_Cys/Ag series are also presented in Figure 3 and reveal significant differences between these two systems. For (Gly)_*n*_Cys/Ag, the reported above S 2p, C 1s, N 1s, and O 1s signals are systematically shifted by ∼0.3–0.4 eV toward lower BE values compared to (Gly)_*n*_Cys/Au SAMs. Whereas in the case of the S 2p peak, this shift can be mainly attributed to changes in the chemical bonding associated with the substrate modification as reported earlier for biphenyl-based thiols,^32,33^ for C 1s, N 1s, and O 1s signals, we attribute this shift in BE to the electrostatic effect induced by the normal component (to the metal surface) of the dipole layer created by the molecules building the monolayer.^34^ It is well known that SAMs form a dipole layer on the metal substrate, which can be used, and engineered, for work function modification of the metal substrate, and therefore for optimizing charge transfer between the metal electrode and organic semiconductor formed on these SAMs *via* tuning the relative position of the metal Fermi level and the highest occupied molecular orbital (HOMO) or lowest unoccupied molecular orbital (LUMO) levels of the organic semiconductor.^35−38^ Such dipole layer based on SAMs consists of two components related to the molecular dipole and the interfacial dipole created by the chemical bonding of the anchoring group to the metal substrate.^35^ For (Gly)_*n*_Cys-based SAMs, the change of the metal substrate induces modification of both components, and therefore, a shift in BE is expected. On the one hand, modification of (Gly)_*n*_Cys chain conformation upon Au → Ag substrate modification, as documented by the IRRAS data and thickness analysis (*vide infra*), changes the respective molecular dipole component. On the other hand, modification of the S–Au bond into a more polarized S–Ag bond^39^ leads to the modification of the interfacial dipole. Importantly, the electrostatic effect reported earlier for alkanethiols formed on Au and Ag substrates shows reversed direction of the BE shift in the C 1s signal upon substrate modification.^33,40^ We have reproduced these results also for the present study, as a reference experiment (see Figure S2). Since the basic nature of chemical bonding with Au and Ag substrates is the same for alkanethiols and (Gly)_*n*_Cys SAMs (*via* formation of the S–Au or S–Ag bond), we can attribute observed difference in electrostatic effects for both types of monolayers mainly to the difference in their molecular backbones and related dipole moments.

**Figure 4:**
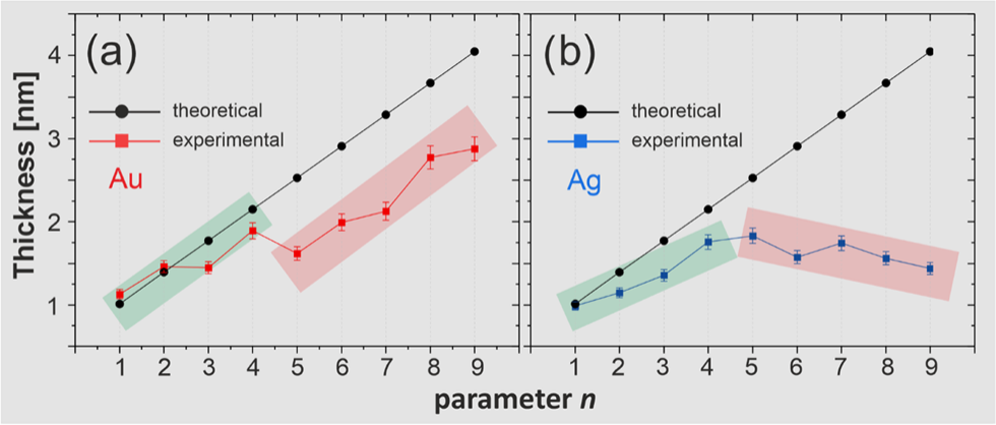
Film thickness for (Gly)*_n_*Cys/Au (a) and (Gly)*_n_*Cys/Ag (b) based on the XPS data analysis (see the text for details) as a function of parameter *n*. The theoretical values indicated in gray correspond to fully extended (Gly)_*n*_Cys molecules. In (a), the green and red bands mark two ranges of *n* values with lower (green) and (higher) tilt angles of β-sheet structures. In (b), green and red bands mark two ranges of *n* values for which the film thickness is growing (green) and descending (red).

**Figure 5:**
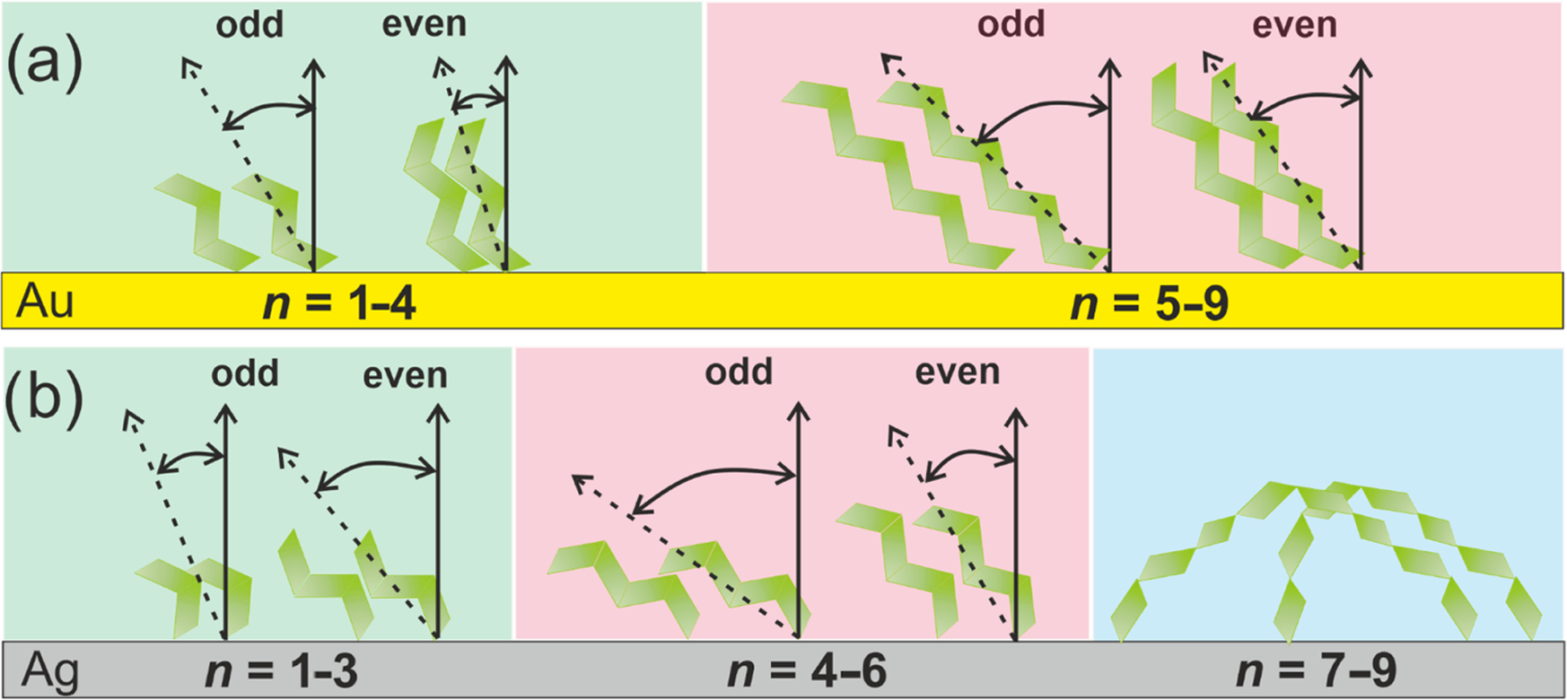
Schematic structural model for (Gly)_*n*_Cys/Au (a) and (Gly)_*n*_Cys/Ag (b) SAMs as a function of parameter *n* (see the text for details).

The *odd–even* structural effect in (Gly)_*n*_Cys SAMs, as well as the huge impact of the metal substrate on their structure, is also well documented by the film thickness analysis based on the ratio of C 1s signal to Au 4f or Ag 3d signals^41^ (Figure S1), considering exponential attenuation of the photoelectron signal with attenuation lengths (λ_C 1s_ = 2.80 nm, λ_Au 4f_7/2__ = 3.11 nm, and λ_Ag 3d_5/2__ = 2.68 nm) calculated on the basis of ref (42), and taking the thickness of hexadecanethiol SAMs on Au and Ag measured in the same experiment as a reference (after correcting the measured intensity of the C 1s signal for (Gly)_*n*_Cys/Au(Ag) SAMs due to the reduced number of C atoms in the molecular chain compared to alkanethiols). The calculated values of *d* obtained for (Gly)_*n*_Cys/Au show a general increase of film thickness with the length of the molecules with, however, an additional *odd–even* variation with parameter *n* (Figure 4a). These results indicate a higher increase of film thickness for *even*-numbered members of the (Gly)_*n*_Cys/Au series associated with their more upright orientation. This information is complementary to the IRRAS data that show the formation of the tilted β-sheet secondary structure but does not solve the question, which *odd* or *even* structures are more upright oriented. The XPS data also confirm secondary structure formation by a significant reduction of the measured film thickness compared to fully extended and normally oriented molecules. Moreover, for *n* = 1–4, the thickness reduction in (Gly)_*n*_Cys/Au is much smaller compared to monolayers with *n* = 5–9 (marked by green and red bands in Figure 4a, respectively). These results are fully consistent with different amplitudes of the *odd–even* behavior in IRRAS data, indicating higher tilt angle and distance between β-sheet secondary structures for *n* = 5–9 compared to *n* = 1–4. The (Gly)_*n*_Cys/Ag SAMs exhibit markedly different results of thickness analysis (Figure 4b). For shorter (*n* = 1–4) members of the series, growth of thickness is observed which is, however, followed by the decay for longer molecules (*n* = 5–9). This decay in film thickness is associated with an overall increase in the respective IRRAS signal. This surprising behavior is correlated, however, with modification of the secondary structure for longer (Gly)_*n*_Cys/Ag SAMs, from the structure close to β-sheet into more looped structures. Such structural modification results not only in the expected film thickness reduction but can also cause an increase of the IRRAS signal, considering the completely different orientation of (Gly) units compared to the β-sheet structure and, therefore, their contribution to the measured IRRAS signal. We note that thickness reduction for long members of the (Gly)_*n*_Cys/Ag series is also well visible in the pronounced S 2p signal for *n* = 9, which is not observed for the analogous monolayer on the Au substrate (Figure 3a). The structural behavior of (Gly)_*n*_Cys/Au and (Gly)_*n*_Cys/Ag SAMs, concluded from the combination of IRRAS and XPS analysis, is schematically summarized in Figure 5 as a function of parameter *n*.

To understand better the possible origin of strong *odd–even* effect reported here, which impacts packing density and tilting of molecules, one can compare it to the well-known *odd–even* structural modifications reported for biphenyl-substituted alkanethiols BP*n*S/Au(Ag) SAMs (CH_3_–C_6_H_4_–C_6_H_4_–(CH_2_)_*n*_–S/Au(Ag), *n* = 1–6).^27,29,43,44^ In BP*n*S/Au(Ag) SAMs, the bulky, and rigid, biphenyl group, attached to the end of alkane chain, forms a steric obstacle which, when tilted (for *n* = even on Au and *n* = odd on Ag), induces strong competition between preferred Au(Ag)–S–C bonding configuration with the metal substrate and two other factors controlling energetics of the system, *i.e.*, packing density and intermolecular interactions.^43^ For (Gly)_*n*_Cys SAMs, such bulky and rigid group is absent, and this system should not show any strong *odd–even* effect similar to simple alkanethiols, where odd–even modification of the aliphatic chain does not influence the packing density and the tilting of molecules (it does influence slightly surface tension^45−47^ and conductance^48,49^ due to reorientation of the top methyl group). However, as a bioinspired system, (Gly)_*n*_Cys SAMs form a secondary structure that is stable enough to build a steric obstacle inducing competition between configuration of the Au(Ag)–S–C bond and the two other factors, *i.e.*, packing density and intermolecular interactions. As a result, for short members of the (Gly)_*n*_Cys series, a reversed *odd–even* effect is observed on Au and Ag substrates. This behavior is similar as for BP*n*S/Au(Ag), although its structural origin is completely different, *i.e.*, it is caused by the secondary structure instead of the primary structure with the bulky biphenyl functional group. For longer members of the (Gly)_*n*_Cys series, this fundamental difference becomes crucial, and the *odd–even* effect is significantly different compared to biphenyl-substituted alkanethiols. This is most probably because the biphenyl functional group provides a fixed structural modification along the series in contrast to the secondary structure whose contribution to the total energetics of the monolayer increases with the parameter *n*. For (Gly)_*n*_Cys SAMs on Au, the increased contribution of the secondary structure leads only to an increased tilt of molecules toward the substrate and thus some modification of the “amplitude” of the *odd–even* effect. For (Gly)_*n*_Cys SAMs on Ag, however, the phase of the *odd–even* effect is reversed for *n* = 4–6 and becomes the same as for the Au substrate, independently of the preferred bonding configuration to the substrate. For longest members of the (Gly)_*n*_Cys series on Ag with *n* =7–9, the *odd–even* effect is modified again due to another contribution to the energetics of the system which is absent for BP*n* SAMs, *i.e.*, the chemical bonding of the top carboxylic group with the Ag substrate by adopting looped conformation, which becomes feasible only for longer molecules.

## 4 Conclusions

In conclusion, our IRRAS and XPS data reveal that the conformation of (Gly)_*n*_Cys oligopeptide SAMs on metal substrates is strongly controlled by the contribution of the secondary structure and the chemical bonding with the metal substrate to the overall energetics of the monolayer. For (Gly)_*n*_Cys/Au SAMs, these contributions lead to a well-defined *odd–even* behavior with higher film thickness and packing density for *even* members of the series. In contrast, for (Gly)_*n*_Cys/Ag SAMs, modification of the molecule–metal bonding configuration, as well as the possibility of an additional chemical bonding between the top carboxylic group and the Ag substrate, leads to a strong dependence of the secondary structure and the *odd–even* effect on the length of the molecule defined by parameter *n*. We would like to stress that although similar strong *odd–even* effects, inducing modification of packing density and orientation of molecules, are well known in nonbiologically inspired SAMs, the origin of *odd–even* effect for (Gly)_*n*_Cys/Ag monolayers is completely different and caused by the (*n* parameter dependent) secondary structure formation instead of primary structure modification with (*n* parameter independent) bulky functional groups. Thus, the present analysis opens up a new class of such *odd–even* effects, which should aid the rational design of biologically inspired SAMs for their biological and electronic applications.

## Supporting Information Available

Table S1 shows the BE and FWHM values for data presented in Figure 3; Figures S1 and S2 show the XPS data for Au 4f and Ag 3d signals obtained for (Gly)_*n*_Cys/Au(Ag) and the C 1s signal for HDT/Au(Ag) samples, respectively (PDF)
