# Supplementary material for: Odd–Even Effect in Peptide SAMs—Competition of Secondary Structure and Molecule–Substrate Interaction
Source: J Phys Chem B. 2021 Sep 23;125(39):10964–71. doi: 10.1021/acs.jpcb.1c06625 (PMC8503877; doi:10.1021/acs.jpcb.1c06625)
Supplement: Supplementary file 1 — jp1c06625_si_001.pdf [file jp1c06625_si_001.pdf]

Supporting Information

for

The Odd-Even Effect in Peptide SAMs – Competition of Secondary Structure and Molecule-Substrate Interaction

Agnieszka Grabarek[1], Łukasz Walczak[2], and Piotr Cyganik[1]*

[1]Smoluchowski Institute of Physics, Jagiellonian University, Łojasiewicza 11, 30-348 Krakow, Poland

[2]Science & Research Division, PREVAC sp. z o.o., Raciborska 61, 44-362 Rogow, Poland

Corresponding Author

*piotr.cyganik@uj.edu.pl

Table S1.

Binding energy (BE) and full width at half maximum (fwhm) parameters for the XPS data obtained for $(Gly)_n$Cys/Au and presented in Figure 3.

Spectrum	Signal	Au				Ag		
		BE [eV] / fwhm				BE [eV] / fwhm		
		CG2	CG6	CG9		CG2	CG6	CG9
C 1s	C-C	284,8 / 1,68	284,8 / 1,92	284,8 / 1,92		284,4 / 1,24	284,4 / 1,34	284,4 1,23
	C-N	286,3/ 1,41	286,3/ 1,73	286,3 / 1,71		285,9 / 1,14	285,9 / 1,22	285,9 / 1,41
	C=O/COOH	288,2 / 1,73	288,2 / 1,61	288,2 / 1,32		287,8 / 1,13	287,9 / 1,17	287,8 / 1,41
N 1s	CONH	400 / 1,82	400 / 1,85	400 / 1,72		399,6 / 1,80	399,6 / 1,72	399,6 / 1,61
	NH_3^+	401,8 / 0,89	-			401,5 / 1,93	401,5 / 1,96	401,5 / 2,47
O 1s	C=O	531,6 / 1,81	531,6 / 1,85	531,6 / 1,71		531,2 / 1,64	531,3 / 1,61	531,2 / 1,63
	COOH	533,2 / 1,86	533,2 / 1,81	533,2 / 1,83		532,8 / 2,07	532,8 / 1,19	532,8 / 0,33
S 2p	$2p_{3/2}$	162,1 / 1,26	162,1 / 1,31	-		161,8 / 1,26	161,8 / 1,28	161,8 / 0,99
	$2p_{1/2}$	163,3 / 1,26	163,3 / 1,31			163,0 / 1,26	163,0 / 1,27	163,0 / 1,00

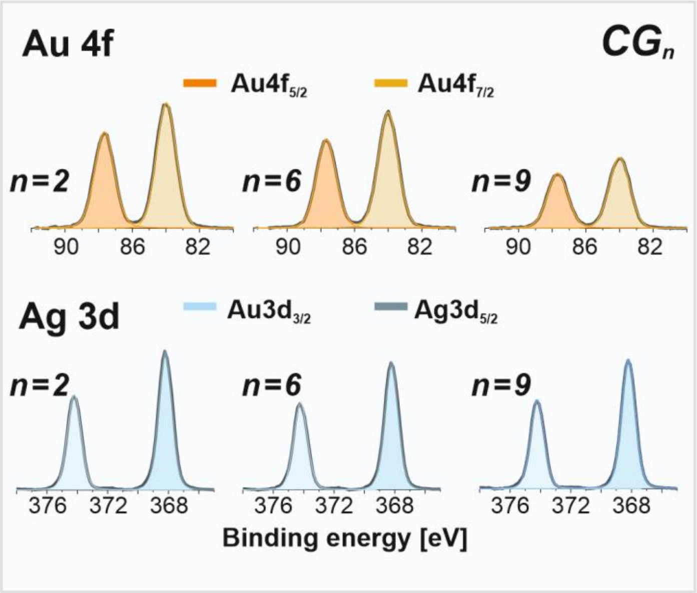

Figure S1. XPS data for (*Gly*)$_n$*Cys*/Au (upper part of the panel) and (*Gly*)$_n$*Cys*/Ag (lower part of the panel) SAMs with *n* = 2, 6 and 9 in binding energy range of Au 4f (a) and Ag 3d(b). Experimental data are indicated by black line.

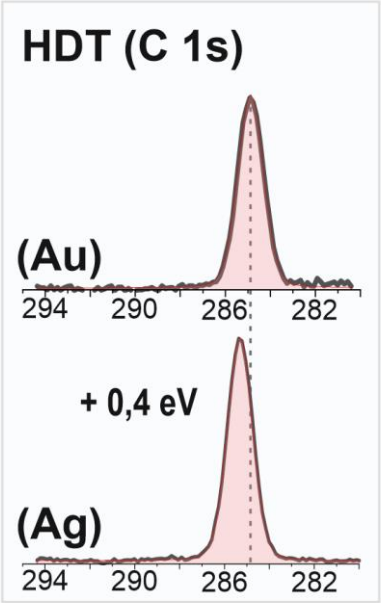

Figure S2. XPS data for hexadecanethiol (HDT) SAMs prepared on Au (upper panel) and Ag (lower panel) substrate in binding energy range of C 1s. Experimental data are indicated by black line. The dashed line marks position of maximum of the signal obtained for HDT/Au SAMs. Next to the signal peaks obtained for HDTs/Ag SAMs the value of the binding energy shift is provided.
